# Development of an ELISA for NNV-Specific Antibody Detection in Grouper Hatcheries in China

**DOI:** 10.3390/vetsci12080754

**Published:** 2025-08-13

**Authors:** Linna Wang, Yongsheng Tian, Zhentong Li, Yishu Qiu, Xiaoyu Ding, Linlin Li

**Affiliations:** 1 Yellow Sea Fisheries Research Institute, Chinese Academy of Fishery Sciences, Qingdao 266071, China; wangln@ysfri.ac.cn (L.W.); lizt@ysfri.ac.cn (Z.L.); yishuqiu97@163.com (Y.Q.); dingxy0503@163.com (X.D.); lill@ysfri.ac.cn (L.L.); 2 Laboratory for Marine Fisheries Science and Food Production Processes, Qingdao National Laboratory for Marine Science and Technology, Qingdao 266000, China

**Keywords:** antibody, ELISA, grouper, nervous necrosis virus

## Abstract

Grouper (*Epinephelus* sp.) is one of the most economically important aquaculture species in China and Southeast Asian countries. Nervous necrosis virus (NNV) infection causes significant mortality in grouper aquaculture. Detection of anti-NNV antibodies using the ELISA method has been reported as effective in preventing vertical transmission of NNV in sea bass and flounder. In this study, IgM antibodies were purified from the serum of groupers and subsequently used to immunize rabbits to obtain a secondary antibody. Additionally, the NNV capsid protein (CP) was cloned, over-expressed, and purified in *Escherichia coli*. Consequently, an ELISA method was developed to detect NNV-specific antibodies in groupers by utilizing purified recombinant NNV capsid protein (CP) and horseradish peroxidase (HRP)-labeled rabbit anti-grouper antibody. This ELISA system was applied in three asymptomatic grouper hatcheries in China, revealing significant variations in antibody levels. Our ELISA method can effectively detect NNV-specific antibodies and is simpler than other methods, making it suitable for subsequent large-scale applications in grouper.

## Simple Summary

Nervous necrosis virus (NNV) is a non-enveloped RNA virus. Following natural infection with NNV, some grouper died from the viral nervous necrosis (VNN) disease, while others remain asymptomatic but can transmit the virus to their offspring. Developing a simple and effective ELISA method can help identify NNV infection in broodstock fish and prevent vertical virus transmission. This study created an ELISA method to detect NNV-specific antibodies in groupers by using purified recombinant NNV capsid protein (CP) and a horseradish peroxidase (HRP)-labeled rabbit anti-grouper antibody. The ELISA system revealed significant differences in antibody levels among three asymptomatic grouper hatcheries in China.

## 1. Introduction

Grouper (*Epinephelus* sp.) is one of the most economically important aquaculture species in China and Southeast Asian countries due to its delicious taste and high market price [1]. *E. Fuscoguttatus* and *E. moara* are two important germplasm resources and farmed grouper varieties in China. They are also important maternal parents in hybrid breeding. Taking advantage of the strong reproductive capacity of *E. Fuscoguttatus*, it was used as the female parent to cross with the fast-growing male grouper, cultivating two new variety of grouper (*E. Fuscoguttatus* ♀ × *E. lanceolatus* ♂ and *E. Fuscoguttatus ♀* × *E. tukula* ♂) with excellent traits of fast growth and strong stress resistance [2,3]. *E. moara* is known for its flavorful meat and high low-temperature tolerance. It has been crossbred with *E. lanceolatus* to create a new grouper variety that exhibits desirable characteristics such as rapid growth and high resilience to stress [4]. In China, these three grouper hybrids make up over 70% of the market share, with Yantai and Hainan Province being two primary grouper hatcheries [1].

However, the grouper farming industry is currently challenged by viral nervous necrosis (VNN), a disease resulting from nervous necrosis virus (NNV) infection. The NNV infection results in significant mortality rates, especially in larval stages, with reported mortalities of up to 100% [2,5]. NNV has been classified into the family Nodaviridae. It is a non-enveloped RNA virus measuring between 25 and 30 nm in size. NNV contains two single-stranded RNA sequences, the RNA1 sequence encodes a 110 kDa RNA-dependent RNA polymerase (RdRp), whereas RNA2 encodes a 42 kDa capsid protein [6]. Over 120 wild and farmed fish species have been reported to be affected by NNV, with red-spotted grouper nervous necrosis virus (RGNNV) being the most prevalent virus [7,8]. NNV mainly targets the central nervous system of fish, causing vacuolation in the brain. Fish infected with NNV frequently exhibit abnormal swimming behavior, impaired swim bladder control and changed body color. The outbreak of VNN during the breeding process of groupers can lead to a low survival rate [8,9]. NNV infection has also been detected in some asymptomatic fish, potentially facilitating viral spread through spawning [9].

Enzyme-linked immunosorbent assay (ELISA) shows good specificity, good sensitivity and high throughput, and it has been used in in vitro diagnostics, epidemiological monitoring, evaluation of immune effects and parental selection breeding [10]. Total levels of IgM and specific antibodies have been reported to be positively correlated with survival rates in Atlantic salmon [11]. NNV asymptomatic infection widely exists in fish aquaculture groups [12,13,14,15,16]. It has been reported that NNV-specific antibodies were detected in 12.5% of *Hyporthodus septemfasciatus* fish [12], and a seroprevalence of 23.8% was detected in the broodstock of barramundi *Lates calcarifer* in Australian hatcheries [13]. The NNV-specific antibody levels generally increased in response to NNV infection, which protected fish from inhibiting NNV replication and prevented the mortality of fish [13]. The spawners were identified as a source of larval infection through ELISA testing for the virus [14]. Detecting anti-NNV antibodies through ELISA can benefit broodstock screening by enabling the monitoring of virus epidemiology without the need to euthanize the fish. The combination of anti-NNV antibody level detection in broodstock and NNV antigen detection in fertilized eggs by PCR effectively prevents the vertical transmission of NNV, increasing the survival rate of offspring against NNV infection [15]. However, the reported ELISA methods for NNV-specific antibodies are cumbersome and costly, thus a simpler and less costly method needs to be established.

In this study, soluble expression of recombinant coat protein of RGNNV was achieved using an *Escherichia coli* BL21 expression system with the pGEX-6P-1 vector. Antibodies from the serum of *E. Fuscoguttatus* were purified using a Protein A column, yielding molecular weights of approximately 70 kDa and 25 kDa. A detection method for anti-NNV antibodies in grouper was developed using an ELISA system based on recombinant NNV capsid protein and HRP-labeled rabbit anti-grouper antibodies. This ELISA system was used to monitor NNV-specific antibodies in three major grouper hatcheries in China, revealing significant variations in antibody levels. Our ELISA method can effectively detect NNV-specific antibodies and is simpler, making it suitable for subsequent large-scale applications in grouper.

## 2. Materials and Methods

### 2.1. Antigen Preparation for ELISA

The coat protein of RGNNV was recombinantly expressed in *Escherichia coli* strain BL21. The gene encoding the RGNNV coat protein was amplified from total nucleic acids from infected *E. fuscoguttatus* using a polymerase chain reaction (PCR) device (nexus GSX1, eppendorf, Shanghai, China). The forward primer, F-exp, which contained the NdeI site (5′-gaattcATGGTACGCAAAGGTGAGAAGAAAT-3′) and the reverse primer, R-exp, which contained the XhoI site (5′-ctcgagGTTTTCCGAGTCAACCCTGGTG-3′), were employed for PCR amplification. The PCR products were ligated to a pMD18-T vector (Sangon Biotech, Shanghai, China) by T4 DNA ligase (Sangon Biotech) and then transformed into *Escherichia coli* DH5a competent cells (Sangon Biotech). Then, the target gene was obtained by enzymatic digestion of the vector with NdeI and XhoI (Takara Bio, Dalian, China), ligated into the NdeI–XhoI site of the pGEX-6P-1 expression vector, and then transformed into BL21 (DE3) competent cells according to the manufacturer’s instructions. The transformed BL21 (DE3) was cultured in a shaker (Zhichu ZQLY-180, Shanghai Zhichu Instrument Co., Ltd., Shanghai, China) at 37 °C and 120 revolutions per minute until the optical density (OD600) reached 0.6–0.8 in Luria–Bertani (LB) broth (1% Bacto tryptone, 0.5% yeast extract, 1% NaCl, pH 7.5) containing 50 mg/mL of ampicillin. The absorbance at 600 nm (OD600) was measured on a microplate reader (PectraMax ICSP1, Molecular Devices, Shanghai, China). Then, 0.5 mM of isopropyl-b-thiogalactoside (IPTG) was added to the culture and incubation was continued at 25 °C for 6 h. These expressing strains were centrifuged in a centrifuge (Avanti JXN-26, BECKMAN COULTER) and resuspended in 10 mM of phosphate buffered saline (PBS, pH 8.0). Then, they were ultrasonicated using an ultrasonic homogenizer (SCIENTZ-IID, Ningbo Scientz Biotechnology Co., Ltd., Ningbo, China) and centrifuged in a centrifuge (5810R, eppendorf, Shanghai, China) at a speed of 6000× *g* for 15 min. The recombinant RGNNV coat protein in the soluble supernatant was then purified with a GST-tag affinity column. Dialysis was performed overnight in an ice bath in a 3000 Da dialysis bag in 10 mM PBS (pH 8.0), and the proteins were analyzed using 12% SDS-PAGE and stored at −20 °C.

### 2.2. The Preparation of Grouper Antibody and HRP-Labeled Rabbit Anti-Grouper Antibody

*E. fuscoguttatus* were farmed in Laizhou city (China). MS-222 was used at 50 mg/L for immersion anesthesia. Blood samples from the caudal vein of 70 fish were collected into labeled tubes, kept at room temperature for 2 h, and then centrifuged at 1500× *g* for 5 min. The serum was pooled to a final volume of 50 mL, diluted with 0.02 M phosphate buffer (PB), and filtered through a 0.22 μM filter membrane. Then, the antibodies were purified from the supernatant using a Protein A column with a 0.1 M pH 3.0 glycine elution buffer and a rapid protein purification system (AKTA Purifier UPC 100, GE Healthcare, Beijing, China). The pH of the elution products was adjusted to neutral using saturated sodium carbonate. The antibodies were concentrated using a 10 kDa ultrafiltration tube and then placed into a dialysis bag for desalination in 0.01 M PBS (pH = 7.4) overnight. After dialysis, the antibodies from groupers were collected and stored at −20 °C. The purity of these antibodies was determined using SDS-PAGE electrophoresis. Protein concentration was determined using the BCA method.

Two Japanese rabbits were immunized four times with a total of 3 mg of grouper antibody to produce antiserum. For the first immunization, 0.5 mg of recombinant protein was mixed with Freund’s complete adjuvant in a 1:1 ratio. The injection was performed once every two weeks. For the second to fourth immunizations, 0.3 mg of the recombinant protein was mixed with 1:1 Freund’s incomplete adjuvant. About one week after the last immunization, blood was collected from the ear vein of the immunized rabbits. After being placed at room temperature for 2 h, the antiserum was separated by centrifugation at 1500× *g* for 5 min. Pre-immunization rabbit serum, irrelevant rabbit antibodies, and PBS were used as negative controls to determine the titer of rabbit polyantibodies. This was achieved by embedding the 2 μg/mL purified grouper antibodies and detecting with HRP-labeled sheep anti-rabbit antibodies (1:10,000 dilution). The rabbit serum was diluted with 0.02 M PBS from 1:2000 to 1:512 K to test its titer, and the antiserum showed a titer of 1:128 k (ODantiserum/ODnegative control ≥ 2.1). The rabbit antiserum was then diluted with 0.02 M PB, filtered using a 0.22 μM filter membrane, and the antibody was purified from the supernatant using a Protein G column with a 0.1 M pH 3.0 glycine elution buffer with a rapid protein purification system (AKTA Purifier UPC 100, GE Healthcare). The pH of the eluted product was adjusted to neutral using saturated sodium carbonate. The antibodies were collected and stored at −20 °C after dialysis in 0.01 M PBS (pH 7.4). The purity of the antibodies was detected using SDS-PAGE electrophoresis, and the protein concentration was measured using the BCA method.

The purified rabbit antibody at 1 mg/mL diluted with 0.01 M PBS (pH = 7.4) was labeled with horseradish peroxidase (HRP) using an HRP TYPE B kit according to the manufacturer’s instructions (MD010, Xingbao Biotechnology Co., Ltd., Suzhou, China). The labeled antibody was stored at −20 °C. Antibody titers before and after labeling were measured by ELISA, and 625 ng/mL of HRP-labeled rabbit antibody was selected for further ELISA detection because it exhibited a relatively good linear relationship.

### 2.3. ELISA System for Detection of Grouper Antibody

The recombinant NNV antigen (1 mg/mL) purified in this study was diluted with a coating solution (50 mM sodium carbonate/sodium bicarbonate, pH 9.6) to 1 μg/mL and added to 96-well plates (Corning, Shanghai, China). ELISA plates were incubated overnight at 4 °C by adding 100 μL of antigen coating solution. The plates were washed once with 200 μL of TBS containing 0.05% Tween 20 (phosphate buffered saline tween 20, PBST) (Solibor Co., Ltd., Beijing, China) and blocked with 5% skim milk powder in PBST at 37 °C for 1 h. After washing in TBST 3 times, 100 μL of the grouper serum to be tested, diluted 1:400, was added and incubated at 37 °C for 1 h. The plate was washed 3 times with TBST and then 100 μL of HRP-conjugated rabbit antibody (625 ng/mL) against grouper IgM was added and incubated at 37 °C for 45 min. After washing in TBST 3 times, 90 μL of 3,3′,5,5′-Tetramethylbenzidine (TMB) single-component color reagent (Solibor Co., Ltd., China) was added to each well and incubated at 37 °C for 20 min. After color development, 50 μL of stop solution (Solibor Co., Ltd., Beijing, BJ, China) was added to each well, and the plate was read using a microplate reader (PectraMax ICSP1, Molecular Devices) at OD450 as soon as possible. Then, 100 μL of PBS was used to replace the grouper serum to be tested for detection baseline absorbance. The fish serum used in the positive control group was taken from one diseased *E. fuscoguttatus* from the Yantai hatchery, which tested positive for NNV infection using specific PCR primers, as described previously [9]. In order to compare and analyze the antigen affinity of recombinant proteins, 1 μg/mL of *E. fuscoguttatus* brain tissue with VNN disease was used for antigen coating and ELISA detection in the positive samples.

### 2.4. Surveillance of NNV-Specific Antibodies in Three Grouper Hatcheries of China

A total of 71 and 88 *E. fuscoguttatus* were sampled from two breeding groups in Hainan Province, respectively. The average body weight was 2510.3 ± 541.6 g and 3674.7 ± 369.5 g, respectively. A total of 390 *E. moara* were sampled from one breeding group in Yantai province. The average body weight was 5846.9 ± 1675.6 g. MS-222 was used at 50 mg/L for immersion anesthesia. Blood samples from the caudal vein of 70 fish were collected into labeled 2.5 mL tubes, kept at room temperature for 2 h, and centrifuged at 1500× *g* for 5 min. The serum was obtained separately and stored at 4 °C; the NNV-specific antibodies in them were detected using the ELISA system. For quality control, PBS and the serum of diseased fish were used as negative and positive controls, respectively, in each separate ELISA plate. Results are presented as the mean ± standard deviation of three replicates. The source of fish is shown in Table 1.

## 3. Results

### 3.1. Antigen for ELISA

**Figure 1:**
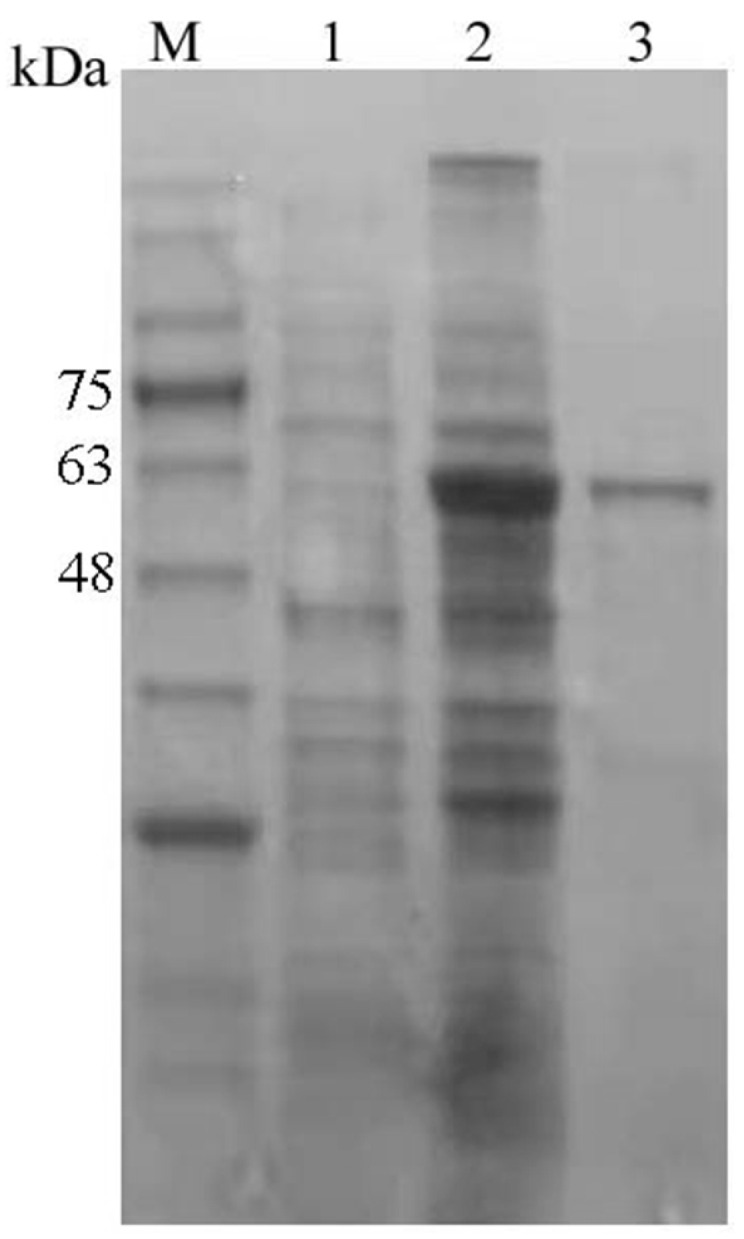
SDS-PAGE of the recombinant coat protein. M, protein markers; 1, the intracellular soluble components without IPTG induction; 2, the intracellular soluble components induced with 0.5 mM IPTG; 3, the purified recombinant coat protein (a band at 60 kDa). (Original image is in Supplementary Materials Figure S1).

The purity of the recombinant coat protein of RGNNV was determined by SDS-PAGE electrophoresis (Figure 1). The purified protein exhibited a single band at approximately 60 kDa, aligning with its theoretical molecular weight. Approximately 150 mg of soluble recombinant protein was obtained per liter of LB culture.

### 3.2. The Preparation of Grouper Antibody and HRP-Labeled Rabbit Anti-Grouper Antibody

**Figure 2:**
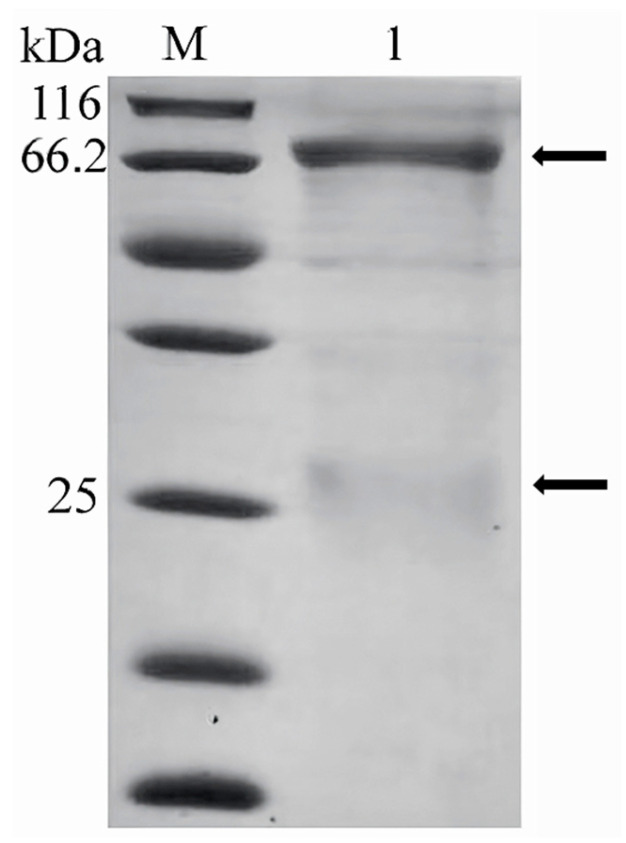
SDS-PAGE of purified grouper serum antibody. M, protein markers; 1, the purified IgM from *E. fuscoguttatus*. The heavy chain displayed a band at 70 kDa, the light chain displayed a band at 25 kDa. (Original image is in Supplementary Materials Figure S2).

**Figure 3:**
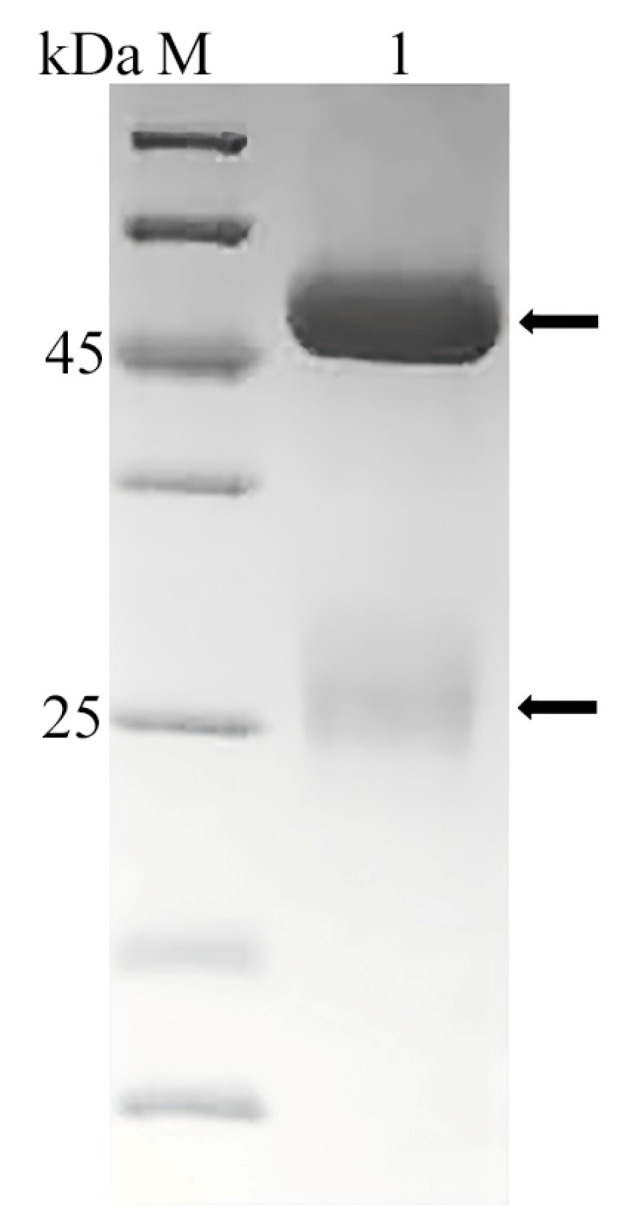
SDS-PAGE electrophoresis of rabbit anti-grouper antibody. M, protein markers; 1, the purified rabbit anti-grouper antibody. The heavy chain displayed a band at 45 kDa, while the light chain exhibited a band at 25 kDa. (Original image is in Supplementary Materials Figure S3).

The purity of the grouper antibody was determined by SDS-PAGE electrophoresis (Figure 2), and the antibody showed a main heavy chain band at about 70 kDa and a weak light chain band at about 25 kDa. A total of 3 mg of antibody was obtained with a concentration of 0.3 mg/mL and a purity of about 80%. After immunization with *E. fuscoguttatus* antibody, the titer of rabbit antiserum was detected to be more than 1:50 K, and a total of 20 mg of rabbit antibody with a purity of over 90% was obtained. SDS-PAGE electrophoresis showed that the antibody exhibited a main heavy chain band at about 45 kDa and a weak light chain band at about 25 kDa by (Figure 3). The OD450 value was 1.02 when the rabbit antibody was detected at 78 ng/mL, and the OD450 value was 1.23 when the HRP labeled with rabbit antibody was detected at 625 ng/mL.

### 3.3. Surveillance of NNV-Specific Antibodies in Three Grouper Farms in China

The baseline absorbance in this study was approximately 0.20 ± 0.04, and the OD450 values of the serum from the diseased *E. Fuscoguttatus* were 0.83 ± 0.06. When 1 μg/mL of *E. fuscoguttatus* brain tissue with VNN disease was used for antigen coating and ELISA detection, the OD450 values of the serum from the diseased *E. Fuscoguttatus* were 0.53 ± 0.06. The results indicated that both ELISA methods, established by these two-antigen embedding approaches, could detect the NNV-specific antibodies in the serum of grouper. The measured values from diseased tissues were slightly lower than those from the recombinant protein, possibly due to the fact that the viral content in the diseased tissues was lower than the content of the embedded recombinant protein.

**Figure 4:**
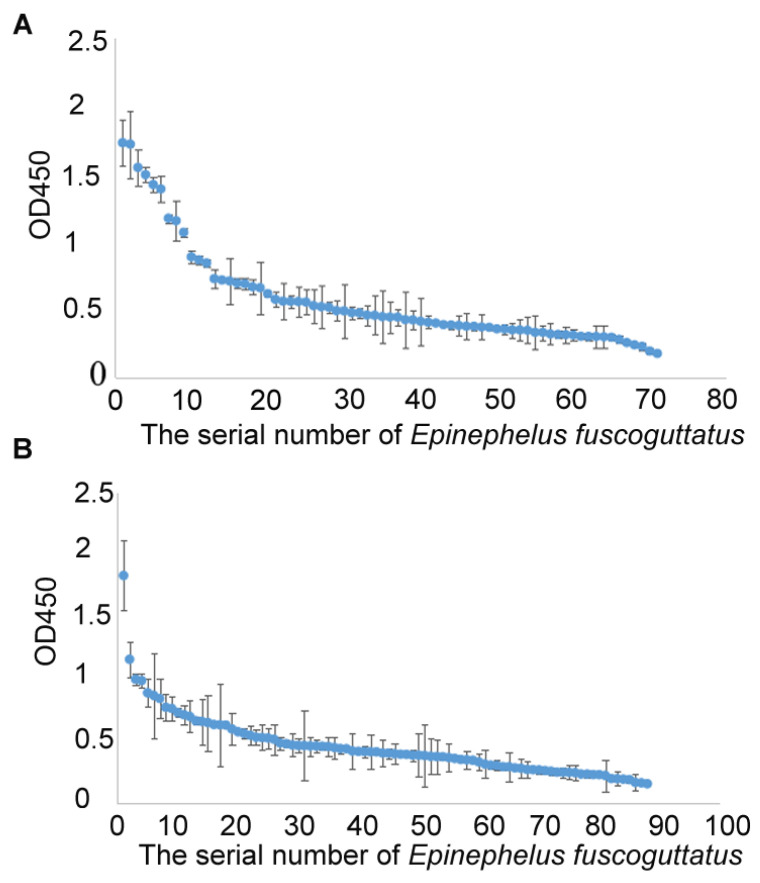
OD450 values from ELISA of sera from *E. fuscoguttatus* populations from Hainan hatchery 1 (**A**) and 2 (**B**) in China. The detected values are arranged from high to low. Results are presented as the mean ± standard deviation of three replicates.

**Figure 5:**
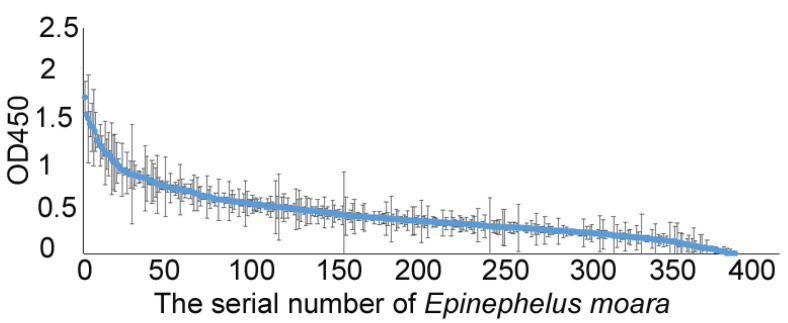
OD450 values from ELISA of sera from *E. moara* populations from a Yantai hatchery in China. The detected values are arranged from high to low. Results are presented as the mean ± standard deviation of three replicates.

Surveillance results of NNV-specific antibodies from three grouper farms in China showed that serum antibody levels vary greatly among individuals. Approximately 28% of the samples from the cultured *E. fuscoguttatus* population 1 showed an OD450 value above 0.60, which was at least three times that of the baseline absorbance (Figure 4A). Approximately 20% of the samples from the cultured *E. Fuscoguttatus* population 2 showed an OD450 value above 0.60 (Figure 4B). Similarly, about 20% of the samples from the cultured *E. moara* population showed an OD450 value above 0.60 (Figure 5). The significant variations in antibody levels indicate that our ELISA method can effectively detect NNV infections, suggesting potential for large-scale applications in grouper.

## 4. Discussion

The grouper industry is currently suffering due to VNN disease. The specific antibody level generated in hosts after virus infection determines the strength of its resistance to disease. Several types of vaccines for VNN disease have been reported, including a formamide-inactivated vaccine, recombinant subunit vaccine and betanodavirus-like pellet vaccine, but no commercial vaccines have been applied to grouper aquaculture yet [17,18,19]. It is difficult to vaccinate larvae fish because their immune systems are not well developed at this stage [20]. Asymptomatic infection has been reported in various fish species, resulting in the production of specific antibodies in breeding groups [12,13,14,15,16]. Thus, ELISA detection of NNV-specific antibodies can reflect the history of NNV infection in the breeding groups. In this study, a detection method for anti-NNV antibodies in grouper serum was established. Surveillance of NNV-specific antibodies in three Chinese grouper farms detected different antibody levels in cultured *E. fuscoguttatus* and *E. moara* populations, which indicated a history of NNV infection in the tested populations.

Several reports have described the ELISA detection method for NNV-specific antibodies in fish. A sandwich ELISA method based on experimental fish sera, NNV virus, anti-NNV rabbit serum and HRP-conjugated swine antiserum was used for NNV-specific antibody detection in sevenband grouper *Hyporthodus septemfasciatus*, and NNV-specific antibodies were detected in one out of eight asymptomatic sevenband grouper [12]. An indirect ELISA method for the detection of antibodies against nodavirus in sea bass (*Dicentrarchus labrax*) was described, based on Rabbit IgG against NNV, NNV virus, experimental sera, biotinylated monoclonal antibody to sea bass IgM and avidin horseradish peroxidase conjugate [14]. An ELISA with a recombinant protein of berfin flounder nervous necrosis virus (BFNNV) was used for the detection of antibodies against BFNNV and applied for the selection of brood fish in order to prevent viral vertical transmissions. It was based on purified striped jack nervous necrosis virus (SJNNV) particles or the recombinant coat protein of BFNNV, fish sera, rabbit serum secondary antibody, and HRP-conjugated antibody against rabbit immunoglobulin [15]. Purifying a capture antigen from a viral culture system is more expensive and complex than purifying it from a recombinant protein system. Therefore, using a recombinant protein is preferable for the ELISA system to detect antibodies against NNV. Although the soluble BFNNV coat protein was obtained from recombinant expression, the amount of soluble protein was much less than the precipitated part. In this study, the recombinant coat protein of RGNNV mainly exists as soluble protein, and about 150 mg of soluble recombinant protein can be obtained per liter of LB culture, which reduces costs. A comparison of NNV-specific antibody ELISA detection methods is shown in Table 2; the results show the ELISA method established in this study is simpler and less costly.

Antibodies from *E. akaara* and *E. awoara* were purified using a Protein A column. The *E. akaara* antibody showed a main heavy chain band at about 74 kDa and two weak light chain bands at about 47 kDa and 27 kDa, as revealed by SDS-PAGE analysis. The *E. awoara* antibody showed a main heavy chain band at about 77 kDa and a weak light chain band at about 31 kDa [21,22]. In this study, the purified antibody from *E. Fuscoguttatus* exhibited a main band at about 70 kDa and a weak band at about 25 kDa. The difference in molecular weight might be attributed to differences in protein markers or species-specific characteristics.

ELISA has been used for VNN diagnosis and to avoid the vertical transmission of viruses. The selection of disease-resistant parents is generally carried out by selecting virus-free individuals as parents through antigen PCR and antibody ELISA detection technology [15]. Indeed, high antibody levels can be passed on to offspring, protecting larvae from horizontal transmission of viruses in the environment. The high NNV-specific antibody level in fish may either be a protective response under NNV infection or residue after recovery from NNV infection. Indeed, fish with high antibody levels which have recovered from infection and no longer carry NNV are also suitable for use as parent fish in breeding. In addition, immune traits have shown moderate-to-high heritability, and breeding for disease resistance according to immune indexes is a kind of indirect selective breeding [22,23,24,25]. An efficient NNV-specific antibody level detection technology is meaningful for in vivo virus detection and indirect selective breeding, although the effectiveness of this ELISA technology in disease resistance breeding varies across different studies [13,15]. The association between the NNV-specific antibody level of grouper and the occurrence of VNN disease in their progeny is not clear. In addition, cross-reactivity between different fish species and sensitivity in different age groups were not analyzed in this study. Thus, more research may be needed to clarify these links in future.

## 5. Conclusions

In this study, an NNV-specific antibody detection technology for grouper was established using an ELISA method based on recombinant NNV capsid protein and HRP-labeled rabbit anti-grouper antibody. Using this ELISA system, the NNV-specific antibodies in three grouper hatcheries in China were surveilled. Parent fish with varying levels of anti-NNV antibodies can be effectively detected using this simpler ELISA method, making it suitable for subsequent large-scale applications in grouper.

## Figures and Tables

**Table 1:** Source of fish in this study.

Source of Fish	Species	Sample Number	Average Weight	Purpose
Yantai hatchery	*E. Fuscoguttatus*	70	-	Preparation for secondary antibody
Hainan hatchery1	*E. Fuscoguttatus*	71	2510.3 ± 541.6 g	Surveillance of NNV-specific antibodies
Hainan hatchery2	*E. Fuscoguttatus*	88	3674.7 ± 369.5 g	Surveillance of NNV-specific antibodies
Yantai hatchery	*E. Moara*	390	5846.9 ± 1675.6 g	Surveillance of NNV-specific antibodies

**Table 2:** Comparison of NNV-specific antibody ELISA detection methods.

Reports	Species	ELISA System	Recombinant Expression System	The Chromogenic Agent	Detection	Characteristics
[12]	*Hyporthodus septemfasciatus*	(Experimental fish sera, NNV suspension, anti-NNV rabbit serum, HRP-conjugated swine antiserum against rabbit Ig)	-	o-phenylenediamine	OD492	Cumbersome and toxic
[14]	*Dicentrarchus labrax*	(Rabbit IgG raised against nodavirus, experimental fish sera, biotinylated monoclonal antibody against sea bass IgM, avidin horseradish peroxidase conjugate)	-	o-phenylenediamine	0D492	Cumbersome and toxic
[15]	*Verasper rnoseri*	(recombinant coat protein of BFNN, fish serum, rabbit serum against barfin flounder IgM, HRP-conjugated antibody against rabbit Ig)	PET-25b (+); insoluble NNV capsid protein	o-phenylenediarnin	OD492	cumbersome and toxic
[13]	*Lates calcarifer*	(sheep anti-NNV primary antibodies, semi-purified NNV, experimental fish sera, rabbit anti-Australian bass antibodies, donkey anti-rabbit horseradish peroxidase (HRP) conjugate)	-	o-phenylenediarnin	OD492	Cumbersome and toxic
This study	*E. Fuscoguttatus*	(recombinant coat protein of RGNNV, experimental fish sera, HRP-conjugated rabbit antibody against grouper IgM)	pGEX-6P-1; soluble NNV capsid protein	TMB	OD450	Simpler, less costly and safe

## Data Availability

Data are available on request from corresponding authors.

## Supplementary Materials

The following supporting information can be downloaded at: https://www.mdpi.com/article/10.3390/vetsci12080754/s1, Figure S1, Original SDS-PAGE image of the recombinant coat protein. Figure S2, Original SDS-PAGE image of purified grouper serum antibody. Figure S3, Original SDS-PAGE image of rabbit anti-grouper antibody.
