# Supplementary figures and images for: Development of an ELISA for NNV-Specific Antibody Detection in Grouper Hatcheries in China
Source: Vet Sci. 2025 Aug 13;12(8):754. doi: 10.3390/vetsci12080754 (PMC12390348; doi:10.3390/vetsci12080754)

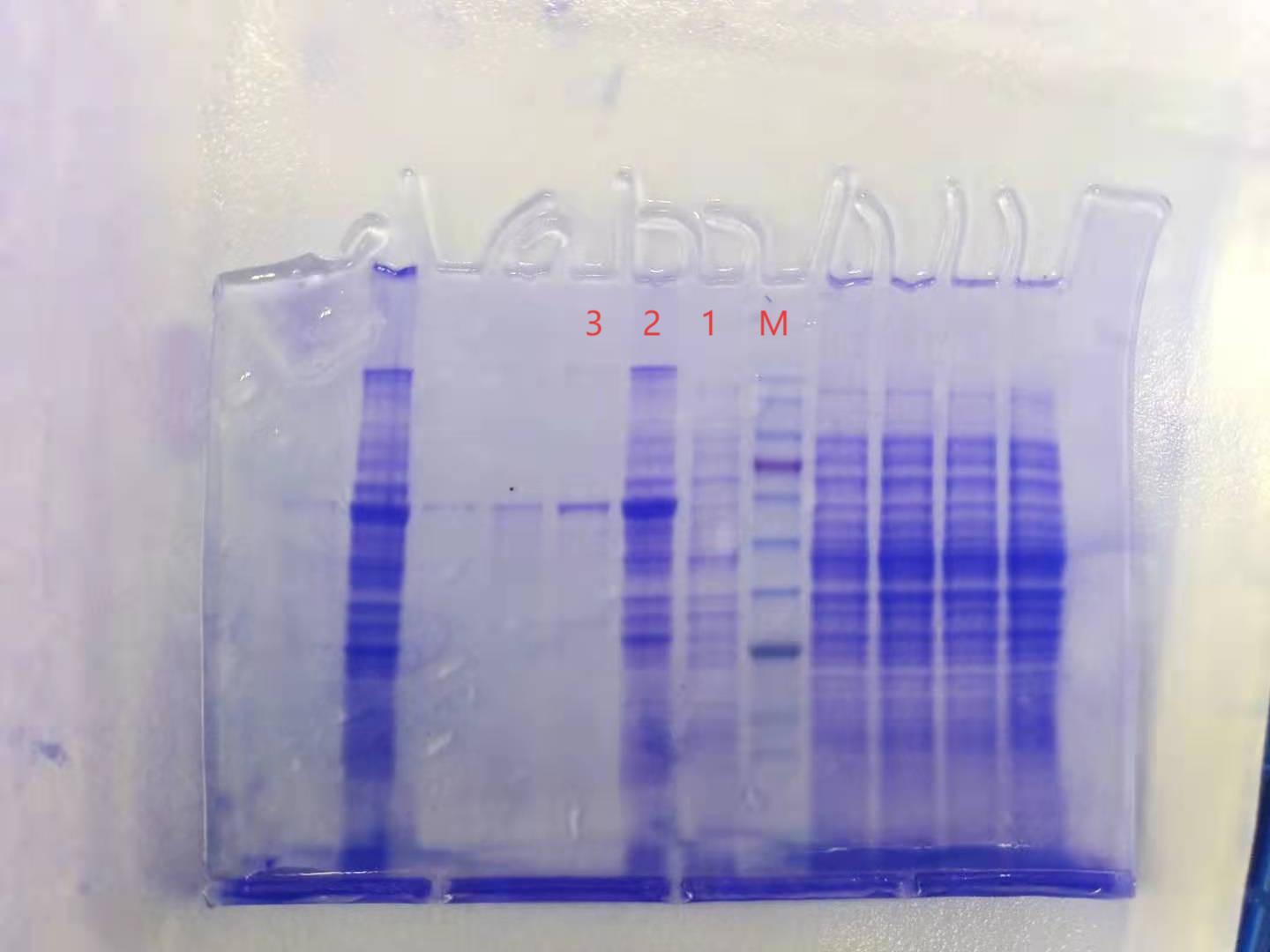

Supplement: Supplementary file 1 [file vetsci-12-00754-s001.zip › Original Images/Figure S1.jpg]

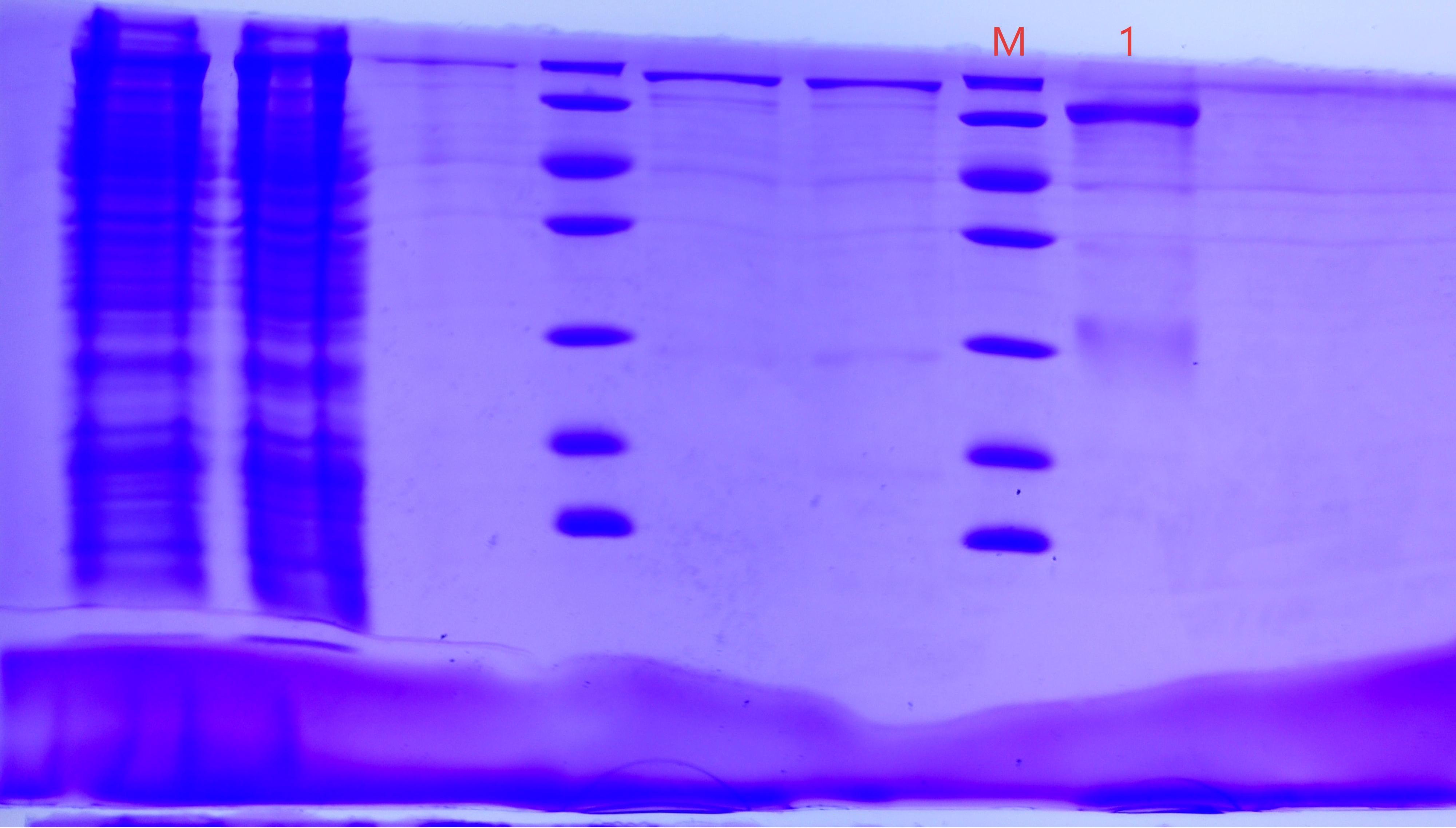

Supplement: Supplementary file 1 [file vetsci-12-00754-s001.zip › Original Images/Figure S2.jpg]

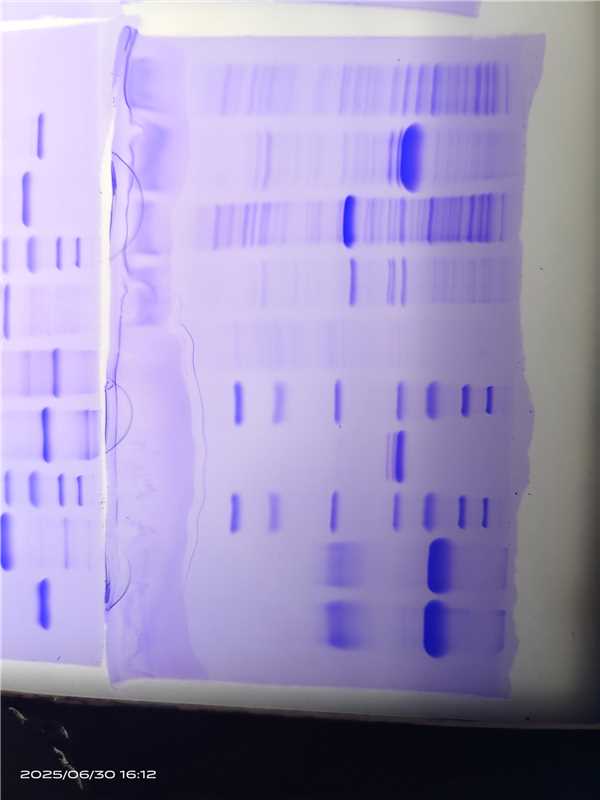

Supplement: Supplementary file 1 [file vetsci-12-00754-s001.zip › Original Images/Figure S3.jpg]
